# Numerical Prediction on the Impact Resistance of UHMWPE Flexible Film Against Hypervelocity Particles

**DOI:** 10.3390/polym18030369

**Published:** 2026-01-29

**Authors:** Hao Liu, Zhirui Rao, Chen Liu, Hao Wang, Jiangfan Zhang, Yifan Wang, Vladimir Simonov

**Affiliations:** 1 School of Mechanical Engineering, Xi’an Jiaotong University, Xi’an 710049, China; drhaoliu@xjtu.edu.cn; 2 School of Physics, Harbin Institute of Technology, Harbin 150001, China; m18345543049@163.com; 3 Laboratory for Space Environment and Physical Sciences, Harbin Institute of Technology, Harbin 150001, China; 4 Collage of Automobile and Traffic Engineering, Nanjing Forestry University, Nanjing 210037, China; zhangjiangfan@njust.edu.cn; 5 Department of Mechanical Engineering Technologies, Bauman Moscow State Technical University, Moscow 105005, Russia; wangyf_wangyf@163.com; 6 First Experimental Design Bureau LLC, Taganrog 347900, Russia; mr.symonov@yandex.ru

**Keywords:** UHMWPE, flexible film, hypervelocity impact, numerical simulation, damage morphology, protective performance

## Abstract

Ultra-high-molecular-weight polyethylene (UHMWPE) thin films are considered promising shielding materials against hypervelocity microparticle impacts in space environments. In this study, a finite element-smoothed particle hydrodynamics (FEM-SPH) adaptive coupling simulation method was developed to reveal the damage mechanisms of UHMWPE films impacted by alumina (Al_2_O_3_) particles with a diameter of 10 μm. A 100 μm thick single-layer UHMWPE film was subjected to normal impacts at velocities ranging from 1 to 30 km/s. The morphology and characteristics of craters formed on the film surface were analyzed, revealing the velocity-dependent transition from plastic deformation to complete perforation. At 10 km/s, additional oblique impact simulations at 30°, 45°, 60° and 75° were performed to assess the effect of impact angle on damage morphology. Furthermore, the damage evolution in double-layer UHMWPE films was examined under impact velocities of 5, 10, 15, 20 and 25 km/s, showing enhanced protective performance compared to single-layer films. Finally, the critical influence parameters for UHMWPE failure were discussed, providing criteria for evaluating the shielding limits. This work offers computational methods and predictive tools for assessing hypervelocity microparticle impact and contributes to the structural protection design of spacecraft operating in the harsh space environment.

## 1. Introduction

The long-term operation of spacecraft in near-Earth or deep space environments is severely threatened by hypervelocity impacts (HVIs) from space debris and micrometeoroids. As the number of space missions increases and satellite constellations expand, the density of orbital debris has risen sharply, which significantly increases the risk of catastrophic collisions [1,2]. Statistical data indicate that more than 100 million debris fragments with diameters larger than 1 mm exist in Earth’s orbit, and their impact velocities generally range from 1 km/s to over 20 km/s [3,4]. Even micrometer-sized particles can cause severe erosion, penetration or secondary fragment ejection when impacting thin-walled spacecraft structures, thereby posing serious challenges to spacecraft safety [5,6]. Therefore, research on spacecraft shielding against HVIs has become one of the key issues in the field of space environment and spacecraft safety engineering.

At present, Whipple shields, which consist of a bumper and a rear wall, represent the most widely applied spacecraft protective structure. The mechanism is based on fragmenting and dispersing incoming projectiles to reduce local damage on the main wall [7,8]. However, Whipple shields exhibit limitations such as increased weight and reduced protective efficiency against high-energy or small-diameter particles [9]. To address these issues, researchers have developed new shield structures, such as multi-shock shields, stuffed Whipple shields and shields incorporating advanced materials [10,11]. Compared with traditional metallic materials such as aluminum alloys, advanced polymer materials offer higher specific energy absorption and excellent toughness, making them promising candidates for next-generation lightweight protective shields [12,13,14].

Ultra-high-molecular-weight polyethylene (UHMWPE) is an advanced polymer with high strength-to-weight ratio, outstanding energy absorption capacity and low density, which has attracted considerable attention in recent years [15,16]. Compared with metals and ceramics, UHMWPE exhibits superior impact resistance and excellent tensile properties, and has been widely applied in ballistic protection, body armor and structural components [17,18]. Moreover, the material has been introduced into aerospace protection systems, demonstrating its ability to effectively mitigate hypervelocity particle impacts [19]. Experimental and numerical studies have shown that UHMWPE not only resists plastic deformation and penetration but also redistributes impact loads via its fibrous microstructure, thereby delaying structural failure [20,21]. Consequently, UHMWPE-based films and laminates are increasingly considered as key elements in spacecraft impact shields.

Recent progress in HVI simulation and experimental techniques has further advanced the study of UHMWPE-based protection systems. For instance, Limido et al. [22] analyzed the fracture and energy dissipation mechanisms of UHMWPE under particle impact, confirming its superior toughness compared with aramid fibers. Reyes et al. [23] investigated the dynamic mechanical response of UHMWPE films under impact loading, showing its ability to absorb substantial kinetic energy. Onder et al. [24] employed molecular dynamics simulations to reveal the role of chain alignment and crystalline orientation in resisting crack propagation during HVIs. In addition, multi-layer UHMWPE structures have been studied extensively, demonstrating their enhanced ability to dissipate energy and protect spacecraft walls compared with single-layer configurations [25,26]. Recent studies [27,28] have begun to apply FEM–SPH coupling to higher-velocity impacts (including hypervelocity and hypersonic regimes) and more complex multiscale scenarios. These works propose improved coupling strategies and calibration procedures aimed at enhancing the predictive accuracy of UHMWPE laminates under varying impact velocities and boundary conditions, particularly in terms of failure mechanisms and energy dissipation. Li et al. [27] employed SPH to describe the fragmentation and ejecta of brittle ceramics, while FEM was used to model the deformation and failure of fiber layers and UHMWPE layers. Their analysis focused on the anti-penetration performance and energy absorption mechanisms under different projectile velocities and impact locations. Kartikeya et al. [28] utilized a coupled FEM–SPH numerical framework to simulate the ballistic response of metal/UHMWPE composite armor structures. Their study emphasized interlayer failure, residual penetration energy, and overall protective efficiency of the armor, demonstrating that the simulations can be applied to engineering-oriented protection design and evaluation.

However, challenges remain in accurately predicting crater morphology, penetration depth and critical impact conditions at velocities exceeding 10 km/s [29,30]. Furthermore, the angle of particle incidence plays a crucial role in determining impact damage morphology. At lower velocities, oblique impacts tend to produce elongated craters and partial delamination, while at higher velocities, grazing incidence can lead to severe surface erosion and localized melting [31]. Understanding these mechanisms is essential for evaluating shielding performance in complex orbital environments, where particles often impact at varying angles. Additionally, the interaction of multiple UHMWPE layers introduces more complex damage mechanisms, including interlayer delamination, fiber pull-out and progressive perforation, which require further computational and experimental investigation [32,33,34].

In studies of multilayer and “stuffed” shield configurations, double-layer or multilayer UHMWPE films generally exhibit higher energy absorption and reduced residual damage on the rear wall compared with single-layer structures. This improvement is mainly attributed to the dispersion of fragment clouds between layers, the stepwise dissipation of energy, and the additional frictional and tensile consumption caused by interlayer relative motion [35,36]. Moreover, the coupling between oblique impact and layered structures may induce localized perforation, scratch propagation, or partial delamination, which raises more stringent requirements for verification under the actual assembly state of spacecraft [37].

Although extensive research has been conducted on UHMWPE fiber composites and soft shields against millimeter- to centimeter-scale particles, systematic numerical investigations focusing on ~10 μm Al_2_O_3_ particles impacting thin films across the full velocity spectrum of 1–30 km/s and at multiple incidence angles remain relatively scarce [38,39]. The coupling of microscale particles, hypervelocity impacts, and thin-film structures necessitates a re-examination of several key issues: the scaling evolution of crater morphology, the critical penetration size and velocity thresholds, the lateral tearing and netting effects induced by oblique incidence, and the distinct damage evolution behaviors of double-layer films across different velocity regimes [40,41]. These issues are directly related to the applicability and safety margins of lightweight flexible shielding in future large-scale space structures [42].

In this study, we employ numerical simulations to systematically investigate the penetration and damage mechanisms of UHMWPE thin films under hypervelocity Al_2_O_3_ particle impacts. The simulations consider 10 μm diameter Al_2_O_3_ projectiles impacting 100 μm thick UHMWPE films at velocities ranging from 1 km/s to 30 km/s. The morphology and damage characteristics of impact craters under normal incidence are analyzed, revealing velocity-dependent transitions in damage modes. Furthermore, oblique impact cases at 30°, 45°, 60° and 75° under 10 km/s velocity are examined to assess angular effects on damage morphology. To further evaluate the protective capacity of multi-layer configurations, double-layer UHMWPE films are subjected to impacts at velocities of 5–25 km/s, and the resulting damage features are compared with single-layer structures. Finally, the critical impact conditions for UHMWPE failure are discussed, providing theoretical support for spacecraft structural protection and contributing predictive methods and tools for assessing hypervelocity microparticle damage in space environments.

## 2. Simulation Method for HVI of UHMWPE Film

In this study, the numerical simulations were conducted using LS-DYNA, a widely applied explicit solver that was originally developed to address HVI problems. LS-DYNA provides multiple approaches, including the Lagrangian method, Eulerian method, Arbitrary Lagrangian-Eulerian (ALE) method and Smoothed Particle Hydrodynamics (SPH). In finite element method (FEM) simulations, materials subjected to extremely high strain rates often experience pronounced nonlinear behaviors such as large deformation, fragmentation, splashing and structural fracture, which may cause severe mesh distortion and numerical divergence. The SPH method, based on a mesh-free particle cloud discretization, avoids the dependence on traditional meshes. However, compared with FEM, SPH typically exhibits lower accuracy and computational efficiency. Therefore, this study adopts the finite element-smoothed particle hydrodynamics (FEM–SPH) adaptive coupling method, which ensures both accuracy and robustness against mesh distortion.

**Figure 1:**
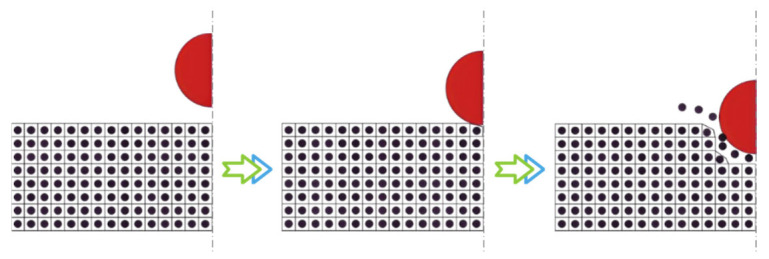
FEM-SPH adaptive coupling: from the initial state to the contact state and then to the erosion state. Black spheres represent SPH particles, with their outer borders indicating virtual interactions, while red spheres denote external particles.

The FEM–SPH coupling is implemented in LS-DYNA through the keyword *DEFINE_ADAPTIVE_SOLID_TO_SPH. In the K file, it is necessary to define the solid parts to be converted into particle regions, the number of SPH particles corresponding to each element, the coupling mode between the particles and the surrounding finite elements, as well as the related parameters. During the initialization stage, the program generates SPH particles at the geometric centers of the specified elements, inheriting the same material properties and computational parameters as the original elements. These particles remain bonded to the parent elements until the failure criterion is reached. Once the elements satisfy the predefined failure conditions, the corresponding meshes are automatically deleted and replaced by SPH particles that inherit their mechanical attributes and geometric volume. The coupling between newly generated particles and the surrounding finite element regions is then automatically established, enabling the continuation of subsequent simulations. The overall conversion and computational process are illustrated in Figure 1.

### 2.1. Simulation Setup for HVIs

**Figure 2:**
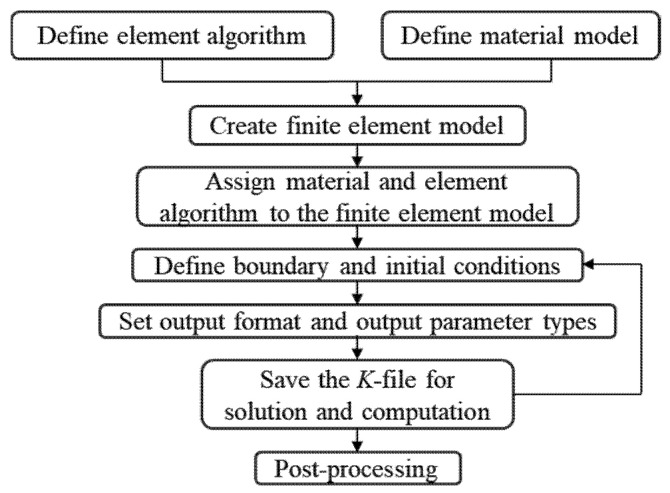
Finite element numerical simulation procedure.

The finite element analysis process mainly includes preprocessing, solving and postprocessing, as shown in Figure 2. Preprocessing steps extend from element algorithm definition to saving the K file. In this work, LS-PrePost was primarily used for model construction, element definition, material property assignment and boundary condition specification.

### 2.2. Algorithm and Material Constitutive Models

The finite element meshes for both UHMWPE film and Al_2_O_3_ particles were modeled using default constant stress solid elements, which are reduced-integration hexahedral elements. This approach reduces the number of integration points, thus simplifying computations and improving efficiency. However, reduced integration elements are prone to hourglass modes. To suppress non-physical hourglass deformation, additional controls were introduced by applying Flanagan–Belytschko viscous control with a parameter value of 0.1. Furthermore, a linear bulk viscosity coefficient of 0.06 and a quadratic bulk viscosity coefficient of 1.5 were introduced to effectively mitigate hourglass effects.

The FEM–SPH adaptive coupling was configured using the *DEFINE_ADAPTIVE_SOLID_TO_SPH keyword, enabling both the Al_2_O_3_ particles and UHMWPE film to convert into SPH particles once the failure criterion was satisfied. The conversion criterion was defined using equivalent plastic strain. Based on extensive trial simulations, the critical equivalent plastic strain was set to 0.4, which showed good consistency between the numerical predictions and experimental observations. The value of 0.4 adopted for the critical equivalent plastic strain was not arbitrarily chosen. This parameter was determined based on relevant literature, available experimental results, and multiple adjustments during the numerical simulation process to achieve stable and reasonable agreement with observed impact behavior. On this basis, the selected value was considered an appropriate and acceptable compromise for the present analyses. In the SPH control setup, once the element reaches the failure criterion, it is removed from the FEM calculation and replaced by SPH particles. The number of particles generated per element was controlled by the parameter NQ = 2, corresponding to eight uniformly distributed SPH particles per element.

To ensure numerical stability and efficiency, smoothing length parameters were carefully defined. In this study, the smoothing length was set to 1.05, with a minimum smoothing length ratio of 0.2 and an upper limit of 2. The B-spline kernel function, widely used in SPH simulations, was employed as the smoothing kernel.

The constitutive behavior of UHMWPE films was described using the Johnson–Cook (J-C) model combined with the Grüneisen equation of state (EOS), defined through the keywords *MAT_JOHNSON_COOK and *EOS_GRUNEISEN. The corresponding parameters are listed in Table 1 and Table 2. The Al_2_O_3_ particles were constructed using the JH-2 constitutive model defined using the keyword *MAT JOHNSON HOLMQUIST CERAMICAS. The parameters are shown in Table 3 below.

### 2.3. JH-2 Constitutive Model

**Figure 3:**
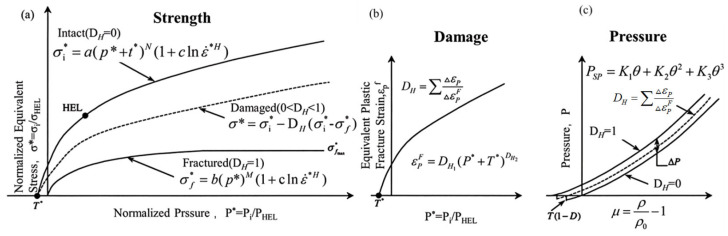
JH-2 Model [48]: (**a**) Strength; (**b**) Damage; (**c**) Equation of state.

In LS-Dyna, the Johnson–Holmquist II model, also known as the JH-2 model, is used to describe the softening behavior of brittle materials during damage. Brittle materials generally have higher strength than plastic materials, making it difficult to obtain their stress–strain curves experimentally. Johnson and Holmquist et al. [47] studied the relationship between hydrostatic stress and equivalent stress, proposing to express hydrostatic pressure as a function of volumetric strain to construct the material’s equation of state; simultaneously, they defined material strength as a function of pressure, thus forming a strength constitutive relationship. In addition, to describe the damage evolution behavior of brittle materials under impact loading, a damage factor *D_H_* is introduced into the model to characterize the effect of damage accumulation on the material’s mechanical properties. The JH-2 model divides the material constitutive model into three parts: strength model, damage model, and equation of state, as shown in Figure 3.

The JH-2 model expresses the equivalent stress of a material as a power-law function of hydrostatic pressure and relates it to the strain rate and damage factor $D_{H}$. The normalized form is given as: $\sigma^{*}=\sigma_{i}^{*}-D_{H}(\sigma_{i}^{*}-\sigma_{f}^{*})$, where $\sigma_{i}^{*}\;={\;a(p^{*}+t^{*})}^{N}(1+c\ln{\dot{\varepsilon }}^{*H})$, $\sigma_{f}^{*}\;={\;b(p^{*})}^{M}(1+c\ln{\dot{\varepsilon }}^{*H})$. In this expression: $\sigma^{*}=\sigma_{i}/\sigma_{HEL}$ denotes the normalized intact strength; $\sigma_{i}$ is the current equivalent stress; $\sigma_{HEL}$ is the equivalent stress at the Hugoniot elastic limit (HEL); a is the intact material strength coefficient; *N* is the intact material strength exponent; b is the fractured strength coefficient; *M* is the fractured strength exponent; *c* is the strain—rate coefficient; $p^{*}=P/P_{HEL}$ is the normalized hydrostatic pressure, $P_{HEL}$ is the pressure at the elastic limit; $t^{*}=T_{H}/P_{HEL}$ is the normalized maximum tensile hydrostatic pressure, $T_{H}$ represents the maximum tensile pressure strength; ${\dot{\varepsilon }}^{*H}={\dot{\varepsilon }}_{H}/{\dot{\varepsilon }}_{H0}$ is the equivalent strain rate, ${\dot{\varepsilon }}_{H}$ is the strain rate, ${\dot{\varepsilon }}_{H0}$ is the reference strain rate.

The JH-2 model adopts the Johnson framework and introduces a damage factor to describe material degradation. The deformation behavior of the material is related to the applied pressure. In the JH-2 model, the equivalent fracture plastic strain $\varepsilon_{P}^{F}$ is expressed as: $\varepsilon_{P}^{F}=D_{H_{1}}{\left(p^{*}+t^{*}\right)}^{D_{H_{2}}}$. Here $D_{H_{1}}$ and $D_{H_{2}}$ are material damage constants.

Damage accumulates progressively with increasing plastic deformation, and the material damage factor $D_{H}$ can be expressed as: $D_{H}=\sum \frac{\Delta \varepsilon_{P}}{∆\varepsilon_{P}^{F}}$, where $\Delta \varepsilon_{P}$ is the equivalent plastic strain accumulated over one integration cycle, and $\Delta \varepsilon_{P}^{F}$ is the equivalent plastic fracture strain under the applied pressure.

In the JH-2 model, the equation of state uses volumetric strain θ to describe the hydrostatic pressure $P_{SP}$, which is divided into compression and tension states. In the compression state: $P_{SP}=K_{1}\theta +K_{2}\theta^{2}+K_{3}\theta^{3}$, and in the tension state: $P_{SP}=K_{1}\theta$. Here, $K_{1}$ is the bulk modulus, $K_{2}$ and $K_{3}$ are equation-of-state constants.

### 2.4. Model Construction and Mesh Generation

The models of Al_2_O_3_ particles and UHMWPE films were established and meshed using LS-PrePost. Since this study involves HVIs at different velocities, angles and particle sizes, the model configurations and mesh densities were adjusted accordingly for each case.

**Figure 4:**
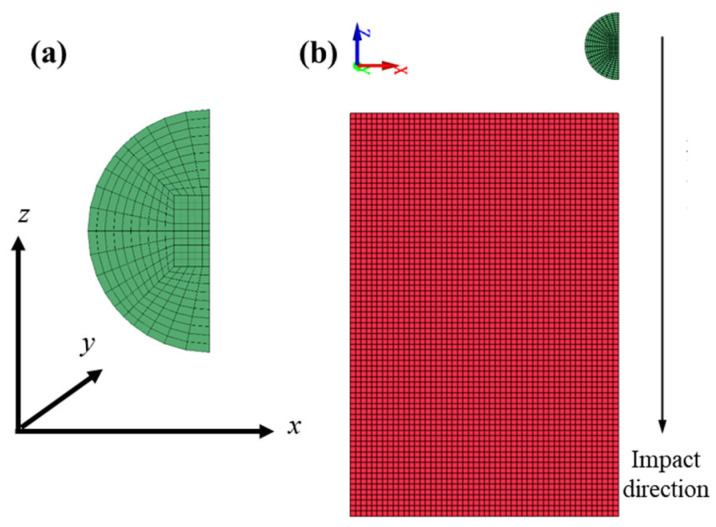
Mesh division for hypervelocity impact at different velocities: (**a**) Meshing of Al_2_O_3_ particle(green color), (**b**) Meshing of Al_2_O_3_ particle and UHMWPE film(red color).

**Figure 5:**
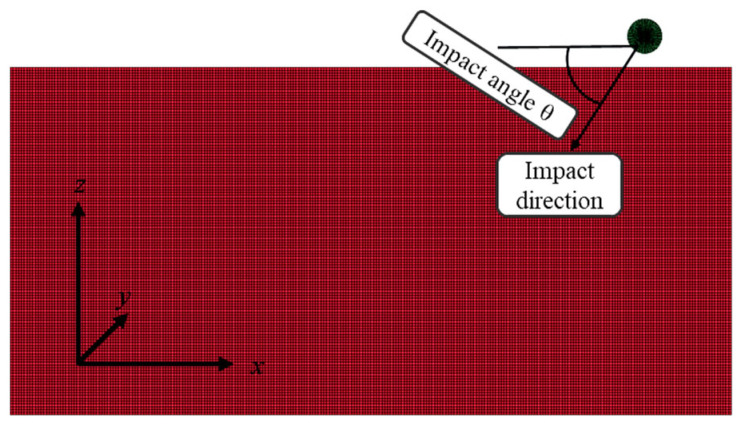
Mesh division for hypervelocity impact at different angles.

**Figure 6:**
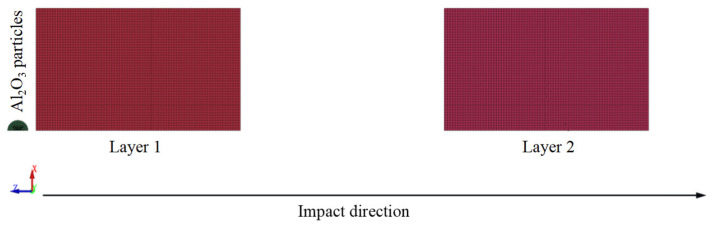
Mesh division for double-layer UHMWPE films under hypervelocity impact.

(1) **HVI simulations at different velocities**: To reduce computational cost, a 1/4 symmetry model was adopted. The Al_2_O_3_ particle had a diameter of 10 μm and was discretized using the sphere solid option in the Shape Mesher with a density setting of 10, resulting in 1750 elements. The UHMWPE film measured 80 μm × 80 μm × 100 μm (length × width × thickness) and was meshed into 0.8 μm cubic elements using the Block Mesher, yielding a total of 703,125 elements. The initial separation distance between the Al_2_O_3_ particle and the UHMWPE film was set to 5 μm, as shown in Figure 4.

(2) **HVI simulations at different incident angles**: In this case, a 1/2 symmetry model was adopted to further reduce computational requirements. The Al_2_O_3_ particle had the same dimensions and meshing method as above but was represented by 3500 elements. The UHMWPE film dimensions were 210 μm × 30 μm × 100 μm, meshed with 0.8 μm cubic elements, resulting in 1,300,000 elements. The initial particle–film distance was also set to 5 μm, as illustrated in Figure 4, where the UHMWPE film is shown in red and the Al_2_O_3_ particle in green. The impact angle was defined as the angle between the projectile velocity vector and the film surface, corresponding to the angle between the impact direction and the x-axis in Figure 5. Numerical simulations were performed for impact angles of 30°, 45°, 60° and 75° at an impact velocity of 10 km/s.

(3) **HVI simulations with different particle sizes**: For simulations involving different Al_2_O_3_ particle diameters, the 0.8 μm element size used for the 10 μm particle was no longer applicable. Therefore, the mesh size was scaled according to the ratio between the given particle diameter and the reference 10 μm particle size to ensure accurate discretization and computational efficiency.
(4) **HVI simulations of double-layer UHMWPE films**: As shown in Figure 6, the double-layer UHMWPE film model followed the same setup as described in Section 2.4 for single-layer films. The difference was that a second UHMWPE film was placed 100 μm beneath the first layer. The second layer had identical dimensions and mesh density as the first, thereby enabling the analysis of layered protection effects under HVI at different velocities.

### 2.5. Boundary Condition Settings

Since a quarter-symmetry model was adopted for the calculation, global constraints (GLOBAL) were first applied to restrict displacements and rotations along the x and y directions on the symmetry planes (in the case of half-symmetry, only the y direction was constrained). In addition, because the FEM–SPH coupling method was employed, the keyword *BOUNDARY SPH SYMMETRY PLANE was used to constrain the SPH particles on the symmetry planes.

Considering that the length and width dimensions of the UHMWPE film are much larger than the diameter of the Al_2_O_3_ particles, preliminary verification indicated that the impact-affected region is relatively small compared with the overall size of the UHMWPE film. Thus, the film in the length and width directions can be regarded as infinitely extended. For computational efficiency, the above-mentioned finite dimensions were selected for the UHMWPE film, while non-reflecting boundary conditions were applied to the two lateral surfaces opposite to the symmetry planes. The keyword *BOUNDARY NON REFLECTING was employed, which effectively prevents shock waves from reflecting at the boundaries, thereby avoiding interference with the simulation results.

## 3. Penetration Mechanism of UHMWPE Film

The validity of the numerical simulation model was verified by comparing the damage characteristics of UHMWPE films observed in experiments with those obtained from numerical simulations [49]. In the simulations, the particle size was set to 10 μm, with impact velocities of 1 km/s and 5 km/s, respectively. Experimental results of UHMWPE films were taken from Case C, including both non-penetration and penetration scenarios.

**Figure 7:**
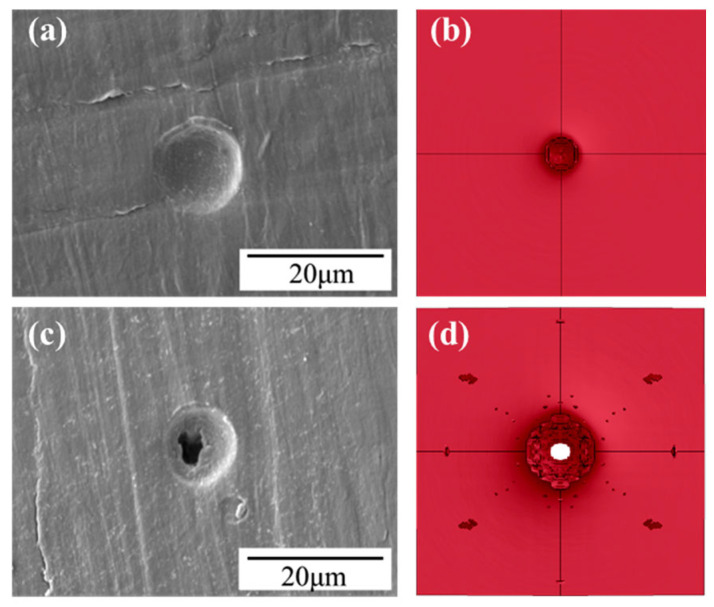
Comparison between numerical simulation and experiment: (**a**) experimental result without penetration, (**b**) surface of UHMWPE film at 1 km/s, (**c**) experimental result with penetration, (**d**) surface of UHMWPE film at 5 km/s.

As shown in Figure 7, when the simulated velocity was 1 km/s, the morphology of the micro-pits was consistent with the experimental observations. At 5 km/s, the UHMWPE film was penetrated, and the morphology of the penetration crater matched well with that observed in Case C. In the 1 km/s simulation, the micro-pit diameter was 14 μm, while at 5 km/s the perforation diameter was 25.54 μm. Both results fell within the statistical range of Case C. Furthermore, at 5 km/s, the maximum penetration diameter differed by 11.2% from the experimental maximum value of 28.76 μm. These results confirm the effectiveness and accuracy of the numerical model.

**Figure 8:**
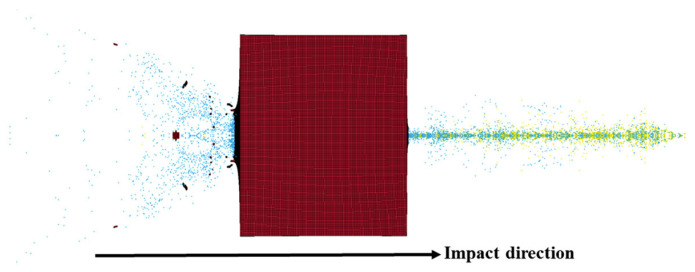
Hypervelocity impact phenomenon at 5 km/s.

Taking the 5 km/s case as an example (Figure 8), the UHMWPE film was completely penetrated. Fragment clouds were generated both on the front (impact) and back sides of the UHMWPE film. The SPH particles in the front-side fragment cloud were mainly generated by the failure of UHMWPE itself, while those at the rear side originated from the combined failure of the UHMWPE film and the Al_2_O_3_ particles. The front-side fragment cloud exhibited a relatively large ejection angle, whereas the back-side cloud was more concentrated.

**Figure 9:**
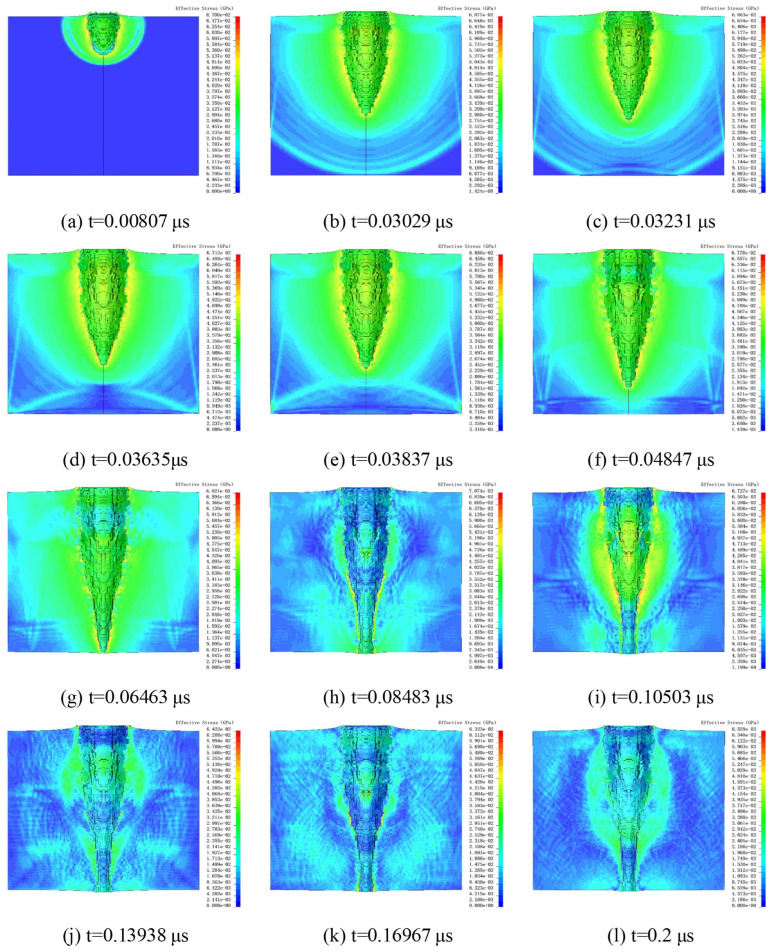
Damage process under 5 km/s impact. The black vertical lines denote the axis of symmetry.

The numerical simulation provided a clear explanation of the damage mechanisms in UHMWPE films under HVIs. Figure 9 shows the simulated process of an Al_2_O_3_ particle impacting the UHMWPE film at 5 km/s. Stress contour plots were used to capture wave propagation within the solid material.

In Figure 9a, the Al_2_O_3_ particle begins to penetrate the UHMWPE film, with the shock wave propagating hemispherically. At t = 0.03029 μs (Figure 9b), the particle had not yet fully penetrated the film, but the stress wave had already reached the rear surface of the UHMWPE film. When the shock wave arrived at the rear surface, reflection waves were formed (Figure 9c). At t = 0.03635 μs, as the reflection wave interacted with the incident shock wave, the stress contours indicated a decrease in stress at the contact region.

As penetration progressed, the stress concentration at the penetration tip continued to extend downward, while the reflection wave dissipated (Figure 9e). Subsequently, the lateral propagation of the incident shock waves was attenuated by the reflection waves, leading to a reduction in stress values. Consequently, the effective penetration diameter decreased after this stage. At t = 0.06463 μs, the UHMWPE film was perforated. By t = 0.08483 μs, most of the Al_2_O_3_ particles had penetrated the film, although residual fragments within the film continued to expand outward, causing the stress concentration to remain highest at the perforation edges. In Figure 9j–l, the stress gradually decreased.

During the penetration process, the crater continued to expand due to inertia. Expansion ceased once the surrounding energy density dropped below the threshold required to induce further material deformation. When the crater size reached its maximum, elastic recovery occurred, leading to a slight contraction of the crater.

## 4. Simulation of UHMWPE Films Under HVI Under Different Conditions

### 4.1. HVI Simulations at Different Velocities

**Figure 10:**
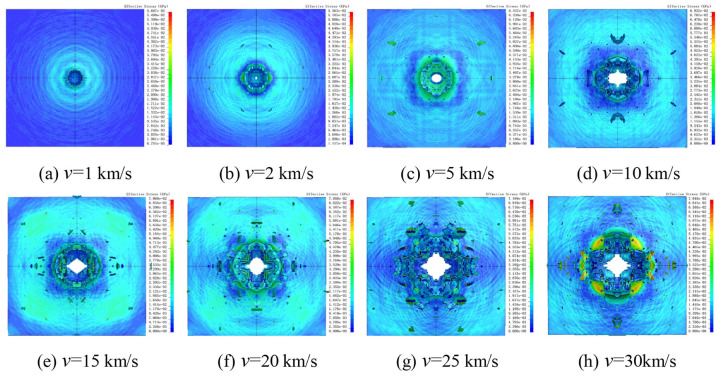
Simulation results at different impact velocities. The black vertical lines indicate the axis of symmetry.

Using the previously described models and material parameters, HVI simulations were performed with Al_2_O_3_ particle velocities ranging from 1 km/s to 30 km/s, with an increment of 5 km/s. Within the 1–5 km/s range, an intermediate velocity of 2 km/s was set to determine the threshold velocity for penetration of 10 μm Al_2_O_3_ particles. Figure 10 shows the damage states and von Mises (V-M) stress contour plots of UHMWPE films under different impact velocities.

At 1 km/s, the Al_2_O_3_ particle failed to penetrate the UHMWPE film, producing a circular impact crater on the surface. The V-M equivalent stress contours indicate a stress concentration at the impact site, gradually decreasing with distance from the impact point. At 2 km/s, the UHMWPE film was perforated, forming a smaller penetration hole in addition to the surface crater, while some surface fragments were ejected without complete failure (Figure 10b). At 5 km/s, as shown in Figure 10c, both the surface perforation and penetration hole enlarged, and the number of surface spall fragments increased.

**Figure 11:**
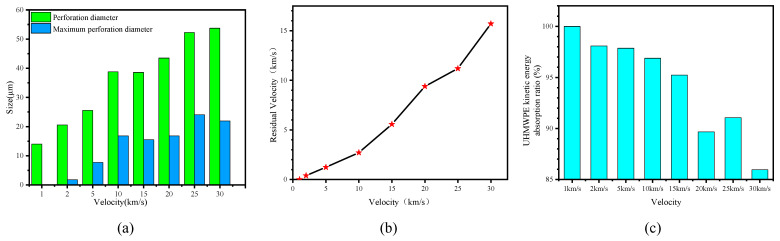
Perforation diameter (**a**), maximum residual velocity (**b**), and absorbed energy ratio of UHMWPE at different velocities (**c**).

Figure 11a presents the minimum surface perforation diameters at different impact velocities. For velocities of 1, 2, 5 and 10 km/s, both the surface and minimum perforation diameters increased with impact velocity. Interestingly, at 15 km/s, the perforation diameters were slightly smaller than at 10 km/s, and at 30 km/s, the minimum perforation diameter was smaller than at 25 km/s. This phenomenon is attributed to two factors: (1) the influence of reflection waves, which reduce the incident shock wave intensity, while the penetration tip remains less affected by the particles, leading to a localized reduction in perforation diameter; (2) elastic contraction after the crater reaches its maximum size.

Figure 11b shows the maximum residual velocities of Al_2_O_3_ particles after penetrating the UHMWPE film. It is observed that the maximum residual velocity increases with impact velocity. Figure 11c presents the kinetic energy absorption rate of the UHMWPE film. At velocities below 20 km/s, the UHMWPE absorbs more than 90% of the projectile’s kinetic energy, with only a small fraction of the Al_2_O_3_ particles retaining substantial residual velocity. As the velocity increases, the energy absorption rate gradually decreases, although it shows an increase at 25 km/s.

### 4.2. HVI Simulations at Different Angles

During in-orbit operations, spacecraft are predominantly struck by space debris at oblique angles. Statistics show that over 90% of collisions occur at incident angles greater than 10°. Compared with normal incidence, oblique impacts are more likely to generate fragment clouds with large scattering angles, with a wider and more complex distribution of secondary fragments, thereby exacerbating erosion and structural damage to spacecraft hulls. Since the impact response and damage mechanisms under non-perpendicular incidence differ significantly from normal impacts, studying HVIs at various angles is critical for improving debris threat assessment accuracy and optimizing protective structure design [50].

**Figure 12:**
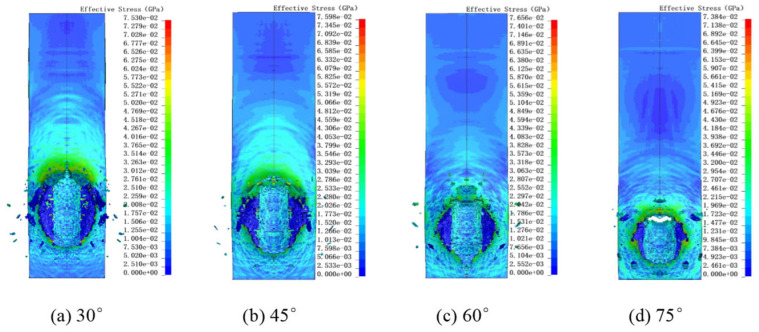
Simulation results at different impact angles. The black vertical lines indicate the axis of symmetry.

**Figure 13:**
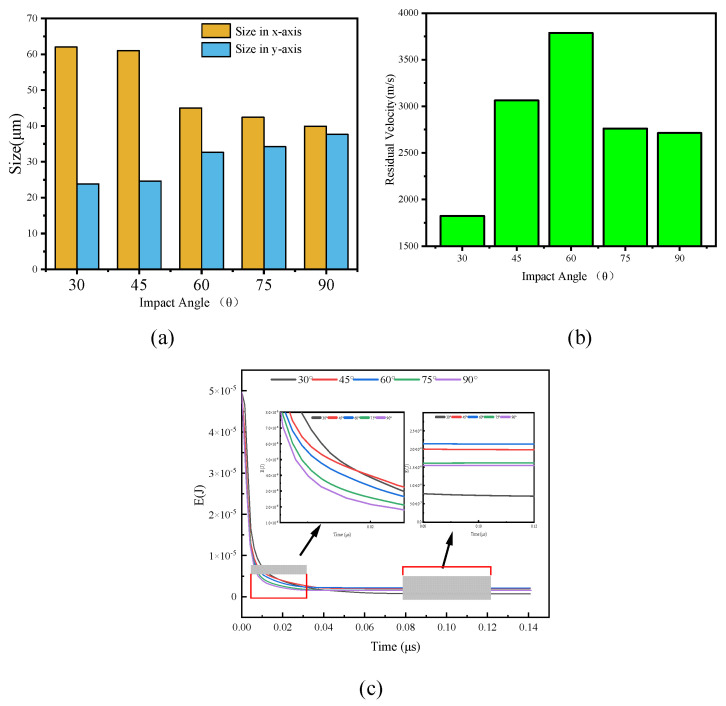
Variations in perforation size, maximum residual velocity, and kinetic energy for 10 km/s impacts at different angles: (**a**) perforation size, (**b**) maximum residual velocity, (**c**) time evolution of kinetic energy.

Figure 12 presents the surface damage and V-M stress distribution of UHMWPE films under different impact angles. The impact craters are generally elliptical. For angles of 30°, 45° and 60°, perforations are not observable from the front side of the film, whereas a small perforation can be observed at 75°. Figure 13a shows the major (Dx) and minor (Dy) axes of surface perforations at different angles. Figure 13b presents the maximum residual velocities of Al_2_O_3_ particles after UHMWPE penetration, and Figure 13c shows the time-dependent kinetic energy of the fragment cloud.

From Figure 13a, as the impact angle decreases, the major axis Dx increases while the minor axis Dy decreases, making the elliptical characteristics of the perforation more pronounced. From Figure 13b, the maximum residual velocity is highest at 60°, while it is relatively similar at 75° and 90°, and minimal at 30°, indicating maximum energy dissipation at the smallest angle. The penetration depth through the UHMWPE film is 100 μm, 103.52 μm, 115.47 μm, 141.42 μm and 200 μm for impact angles of 90°, 75°, 60°, 45° and 30°, respectively. At 30°, the particle penetrates the farthest, consuming the most energy. The small difference of 3.52 μm between 75° and 90° explains the minor difference in residual velocity. The maximum residual velocity occurs at 60°.

Analyzing the time evolution of the Al_2_O_3_ fragment cloud kinetic energy (Figure 13c), at the initial impact (≈0.02 μs), the kinetic energy from largest to smallest corresponds to impact angles of 30°, 45°, 60°, 75° and 90°. At 0.08 μs, when kinetic energy stabilizes, the order is 60°, 45°, 70°, 90° and 30°. Since the minimum distance between Al_2_O_3_ and UHMWPE is 5 μm for all angles, larger impact angles result in shorter particle–film distances. Consequently, at the same time (0.02 μs), 90° impact reduces Al_2_O_3_ kinetic energy most rapidly. When reflection waves interact with the incident shock wave, unloading reduces penetration velocity. Only the bottom of the UHMWPE film produces reflected waves due to the non-reflecting lateral boundary conditions. As the impact angle decreases, the normal component of the incident shock wave decreases, reducing the influence of reflection waves. Therefore, the interaction between penetration depth and reflection wave effects results in maximum residual kinetic energy of the fragment cloud at a 60° impact.

### 4.3. Damage Characteristics of Double-Layer UHMWPE Films Under Different Impact Velocities

**Figure 14:**
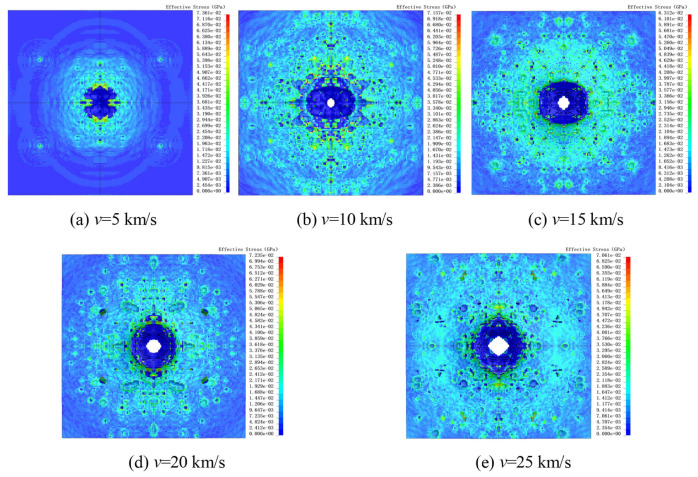
Stress cloud diagrams of surface damage in the second UHMWPE film layer at different velocities. The black vertical lines indicate the axis of symmetry.

Figure 14 shows the surface damage and stress contour distribution of the second-layer UHMWPE film. As seen in Figure 14a, at an impact velocity of 5 km/s, the Al_2_O_3_ fragment cloud failed to penetrate the second-layer UHMWPE film. The fragment cloud produced a large impact crater on the film surface along with minor micro-pit damage, indicating that the double-layer UHMWPE configuration can fully protect against 10 μm Al_2_O_3_ particles at 5 km/s.

**Figure 15:**
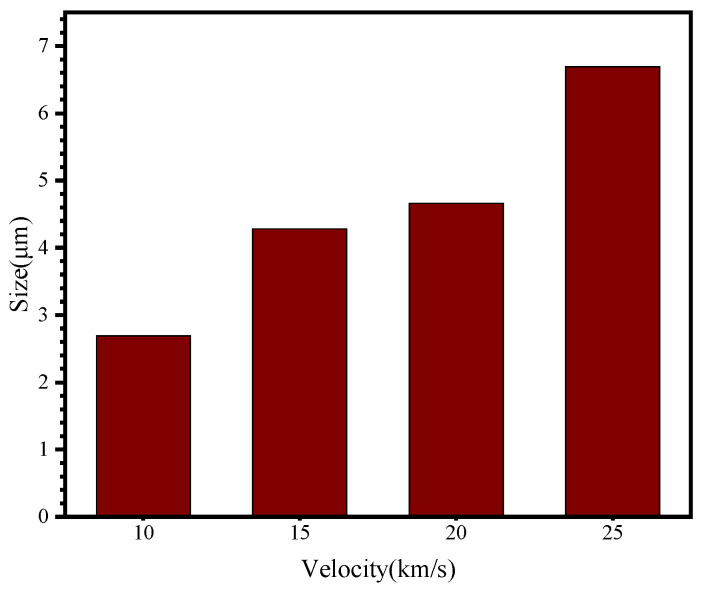
Perforation size of the second UHMWPE film layer at different velocities.

In Figure 14b, when the impact velocity increased to 10 km/s, the second-layer UHMWPE film was penetrated. A small penetration hole appeared on the film surface, while the crater region composed of fragmented material expanded. Figure 15 presents the sizes of penetration holes on the surface of the second-layer UHMWPE film. It can be observed that as the impact velocity increases, the penetration hole size on the second-layer surface gradually enlarges. Compared with the first-layer UHMWPE film (Figure 10a), the penetration size of the second layer is significantly smaller, demonstrating the additional protective effect provided by the double-layer configuration.

## 5. Discussion on the Critical Thickness of UHMWPE Films Under Impact

The study of extreme impact behavior requires a clear definition of the limiting impact condition. The limiting impact condition refers to the scenario in which a projectile of specific size, shape, and material, impacting a protective structure of defined size and material at a given angle and velocity, has a 50% probability of penetrating the structure. This state is defined as the critical state, with the corresponding velocity referred to as the critical impact velocity, and the associated projectile size as the critical impact diameter. Determining the 50% probability in practice requires extensive experimental data, which is nearly impossible to achieve [51]. We admit that actual structural performance may differ substantially due to the complex dynamic effects noted by the reviewers.

**Figure 16:**
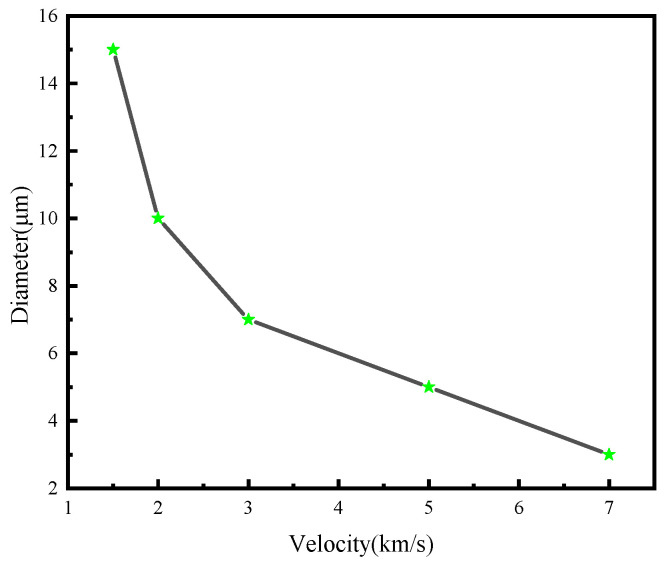
Critical perforation size of UHMWPE film under hypervelocity impact.

In this study, the critical impact velocities of Al_2_O_3_ particles with diameters ranging from 1 to 10 μm were obtained primarily through numerical simulations. By conducting multiple simulations, if a particle does not penetrate at velocity V_1_ but penetrates at velocity V_2_, a velocity between V_1_ and V_2_ is regarded as the critical impact velocity under the given conditions. Figure 16 presents the critical dimensions of UHMWPE films under impact. The results indicate that at a velocity of 1.5 km/s, the critical diameter of Al_2_O_3_ particles required to penetrate the UHMWPE film is 15 μm, whereas at 7 km/s, the critical diameter decreases to 3 μm.

### 5.1. Validation of the Simulation Method

To verify the accuracy of the numerical simulation model, Al_2_O_3_ particles with a diameter of 10 μm were selected, and impact velocities of 1 km/s and 5 km/s were considered. The simulation results are shown in Figure 6. At 1 km/s, the simulated micro-crater morphology closely matches the experimental results, demonstrating good agreement. At 5 km/s, the UHMWPE film is fully penetrated, and the penetration hole morphology in the simulation aligns well with the experimental observations (Experiment C). Specifically, the simulated micro-crater diameter at 1 km/s is 14 μm, and the surface penetration diameter at 5 km/s is 25.54 μm, both falling within the statistical range of the experimental data. The maximum penetration diameter at 5 km/s shows an 11.2% deviation from the experimental maximum value of 28.76 μm. These results confirm that the established simulation model can effectively capture impact deformation, failure, and penetration phenomena, providing a reliable basis for subsequent studies.

### 5.2. Residual Velocity Analysis

**Figure 17:**
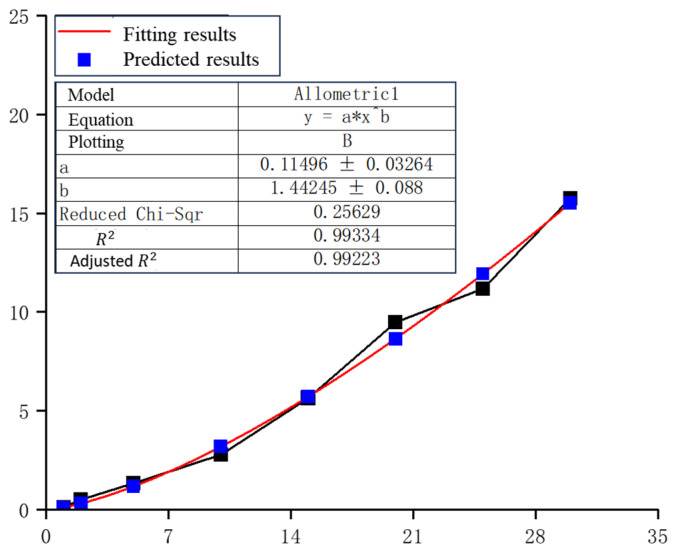
Fitting results of residual velocity. The symbols x and y correspond to $v_{0}$ and $v_{r}$, and fitting parameters a and b equal to 0.115 and 1.442.

Based on the validated simulation method, further parametric studies were conducted. In these simulations, 10 μm Al_2_O_3_ particles impacted single-layer UHMWPE films with dimensions of 80 μm × 80 μm × 100 μm, meshed with 0.8 μm cubic elements. The initial distance between the particle and the film was set to 5 μm. The simulation results, shown in Figure 11b, indicate that the maximum residual velocity of Al_2_O_3_ particles after penetration increases with the impact velocity. Fitting the simulation data yields an empirical relationship between impact velocity *v*_0_ and residual velocity $v_{r}=0.115\cdot v_{0}^{1.442}$ as shown in Figure 17.

According to this relationship, a 30 km/s Al_2_O_3_ particle retains a residual velocity of approximately 15.75 km/s after penetrating a 100 μm single-layer UHMWPE film. Conversely, particles with velocities ≤ 1 km/s cannot penetrate the same film thickness. By iteratively applying the fitted formula, the residual velocities for successive penetrations through 100 μm UHMWPE layers are approximately: 15.75 km/s to 6.126 km/s to 1.570 km/s to 0.220 km/s. Therefore, a 30 km/s particle would require either a 400 μm single-layer UHMWPE film or four stacked 100 μm layers to be fully intercepted.

### 5.3. Effect of Spaced Target Configuration

Further analysis considered the effect of a spaced target configuration, in which two UHMWPE layers are separated by a gap, as illustrated in Figure 14a–c. At impact velocities below 5 km/s, Al_2_O_3_ particles can penetrate the first UHMWPE layer but fail to penetrate the second layer. This effect reduces the required overall UHMWPE thickness compared to a solid single-layer film. For instance, while a 30 km/s particle would nominally require a 400 μm single-layer or four-layer 100 μm film to be intercepted, adopting a spaced target configuration can reduce the required thickness to 300 μm single-layer or three 100 μm layers. This phenomenon is attributed to the deceleration and dispersion of the fragment cloud in the gap, which enhances the energy dissipation before reaching the second layer.

## 6. Conclusions

The paper conducts numerical simulations of the hypervelocity impact on UHMWPE films using the FEM-SPH coupling method, investigating the protective performance and damage mechanisms of UHMWPE under particle impacts at different velocities, as well as its protective performance under impacts at various angles. The main conclusions are as follows:

(1) Under HVI, UHMWPE films generate a cloud of ejected debris on the surface, while the penetrated region forms a concentrated debris cloud. Analysis of the penetration and perforation mechanism indicates that spindle-shaped perforations form during the early stage of impact. As the reflected waves develop, the stress at both sides of the penetration tip decreases, reducing the failure region and the perforation size. Consequently, the Al_2_O_3_ debris cloud from the penetrated UHMWPE film is relatively concentrated.
(2) Single-layer UHMWPE films can effectively protect against 10 μm Al_2_O_3_ particles at 1 km/s, while double-layer UHMWPE films can protect against 10 μm particles at 5 km/s. A 10 μm particle at 2 km/s can penetrate a 0.1 mm single-layer UHMWPE film; however, analysis of the energy absorption shows that UHMWPE films can absorb over 90% of the particle kinetic energy at velocities below 20 km/s, and still absorb over 80% at velocities between 20 and 30 km/s.
(3) Numerical simulations for different impact angles (30°, 45°, 60°, 75° and 90°) reveal that, for the same film thickness, smaller impact angles lead to longer penetration distances. Conversely, at larger angles, reflected waves in the normal direction of the UHMWPE surface have greater influence, while the decay of the shock wave is reduced. The interaction of these effects results in the highest residual energy of the Al_2_O_3_ debris cloud at a 60° impact angle.
(4) The critical penetration size of UHMWPE films was analyzed: for a particle velocity of 1.5 km/s, the critical diameter of Al_2_O_3_ particles to penetrate the film is 15 μm, while at 7 km/s, the critical diameter decreases to 3 μm. Double-layer UHMWPE films can protect against 10 μm particles at 5 km/s. It is predicted that to fully intercept a 30 km/s Al_2_O_3_ particle, a 400 μm single-layer film or four 100 μm layers of UHMWPE are required.

## Figures and Tables

**Table 1:** UHMWPE Parameters [43].

*E* (MPa)	Poisson Rate	*ρ* (g/cm^3^)	*A* (MPa)	*B* (MPa)	*n*	*m*
1080	0.4	0.97	16.11	39.29	0.57	1

**Table 2:** Parameters of Mie–Grüneisen Equation [44].

Material	*Γ*	*C*_0_ (km/s)	*S*_1_
UHMWPE	1.64	2901	1.49

**Table 3:** Al_2_O_3_ Parameters [45,46].

Constants	Value	Constants	Value
*ρ* (g/cm^3^)	3.84	Maximum tensile pressure strength, *T* (GPa)	0.262
Shear modulus, *G* (GPa)	135	Pressure at HEL, *P*_HEL_ (GPa)	1.46
Intact strength coefficient, *a*	0.93	Damage coefficient, *D*_H1_	0.005
Fracture strength coefficient, *b*	0.31	Damage exponent, *D*_H2_	1.0
Strain rate coefficient, *c*	0.007	Bulk modulus, *K*_1_ (GPa)	220
Intact strength exponent, *N*	0.64	Pressure coefficient, *K*_2_ (GPa)	0
Strength Exponent, *M*	0.6	Pressure coefficient, *K*_3_ (GPa)	0

## Data Availability

The original contributions presented in this study are included in the article. Further inquiries can be directed to the corresponding authors.
